# Habitat imaging combined with multimodal analysis for preoperative risk stratification of papillary thyroid carcinoma

**DOI:** 10.1186/s13244-025-02145-9

**Published:** 2025-12-02

**Authors:** Jia-Wei Feng, You-Long Zhu, Lu Zhang, Yu-Xin Yang, An-Cheng Qin, Shui-Qing Liu, Yong Jiang

**Affiliations:** 1 Department of Thyroid Surgery, The Third Affiliated Hospital of Soochow University, Changzhou First People’s Hospital, Changzhou, China; 2 Department of Gastrointestinal Surgery, Southeast University Affiliated Xuzhou Central Hospital, Xuzhou, China; 3 Department of Ultrasound, Ruijin Hospital, Shanghai Jiao Tong University School of Medicine, Shanghai, China; 4 Department of Thyroid Surgery, Suzhou Municipal Hospital, The Affiliated Suzhou Hospital of Nanjing Medical University, Suzhou, China; 5 Department of Ultrasound, The Third Affiliated Hospital of Soochow University, Changzhou First People’s Hospital, Changzhou, China

**Keywords:** Papillary thyroid carcinoma, Habitat imaging, Risk stratification, Machine learning, Multimodal imaging

## Abstract

**Objective:**

To develop a comprehensive preoperative risk stratification model using habitat imaging combined with multimodal analysis for identifying low-risk papillary thyroid carcinoma (PTC) patients suitable for active surveillance.

**Materials and methods:**

This multicenter study analyzed 1215 patients with pathologically confirmed PTC from four Chinese institutions. Habitat imaging analysis was performed on preoperative CT and ultrasound images using K-means clustering and supervoxel segmentation. Radiomic features were extracted from ultrasound habitats using PyRadiomics, while multi-scale index (MSI) features were extracted from CT habitats. Clinical characteristics and immunological markers were identified through multivariate logistic regression. Six machine learning algorithms were evaluated with three fusion strategies to integrate imaging features with clinical data.

**Results:**

Four ultrasound habitats and five CT habitats were identified. Ultrasound Habitat 2 achieved an AUC of 0.92 in training and 0.80–0.92 in validation. CT habitat analysis using MSI features achieved an AUC of 0.93 in training and 0.88–0.92 in validation. The optimal ensemble fusion model integrating CT-derived MSI features, ultrasound habitat characteristics, clinical parameters (chronic lymphocytic thyroiditis and tumor size) and immunological markers (platelet-to-lymphocyte ratio) achieved an AUC of 0.98 in training, 0.95 in internal validation, and 0.95–0.99 across external validation cohorts, with accuracy exceeding 0.88 in all validation sets.

**Conclusion:**

Habitat imaging combined with multimodal analysis provides superior preoperative risk stratification for PTC, enabling personalized treatment planning and identification of low-risk patients suitable for active surveillance while potentially reducing unnecessary surgical interventions.

**Critical relevance statement:**

Habitat imaging combined with multimodal analysis provides superior preoperative risk stratification for papillary thyroid carcinoma, enabling personalized treatment decisions and reducing unnecessary surgical interventions.

**Key Points:**

- Current papillary thyroid carcinoma (PTC) risk stratification relies on postoperative pathology, limiting preoperative treatment planning.
- Multimodal habitat imaging achieved exceptional performance across validation cohorts.
- This framework enables personalized treatment planning and identifies low-risk patients for active surveillance.

**Graphical Abstract:**

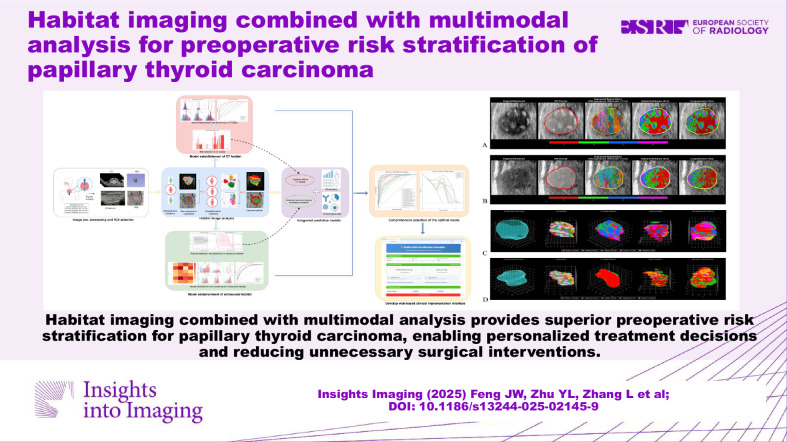

## Introduction

Papillary thyroid carcinoma (PTC) is the most common thyroid malignancy, accounting for approximately 85–90% of all thyroid cancers [1]. Despite generally favorable outcomes, PTC demonstrates significant heterogeneity in clinical behavior and prognosis. Current risk stratification systems, such as the American Thyroid Association (ATA) guidelines [2], rely primarily on postoperative pathological findings, including tumor size, extrathyroidal extension, and lymph node metastasis status. These conventional approaches require surgical intervention before risk assessment, limiting preoperative treatment planning and potentially leading to inappropriate therapy selection for individual patients.

Traditional radiomics approaches extract features from entire tumor regions, assuming homogeneous characteristics throughout the lesion [3]. This assumption often fails to capture complex spatial variations within tumors. Habitat imaging, defined as the computational segmentation and analysis of tumor subregions with distinct imaging phenotypes that reflect underlying biological heterogeneity, addresses this limitation by identifying spatially distinct tumor subregions based on quantitative imaging characteristics [4].

The integration of multimodal preoperative data offers promising opportunities for accurate risk prediction. CT provides anatomical information about tumor morphology and structural characteristics. Ultrasonography offers a detailed assessment of echogenicity, calcifications, and tumor boundaries. Clinical features and immunological markers provide additional biological insights that complement imaging findings. Machine learning techniques can effectively integrate these diverse data sources to identify complex patterns associated with different risk levels [5].

This study aimed to develop a comprehensive preoperative risk stratification model for PTC using habitat imaging combined with multimodal analysis. Our approach integrates CT and ultrasound-derived habitat features with clinical characteristics and immunological markers to accurately predict postoperative risk categories before surgical intervention, enabling personalized treatment planning for individual patients.

## Materials and methods

### Study design and patient population

This multicenter study was approved by the institutional review boards of Changzhou First People’s Hospital, Shanghai Ruijin Hospital, Suzhou Municipal Hospital, and Xuzhou Central Hospital (Approval No. 2019-N-97; 2020-N-65; 2022-N-192; 2024-N-221), and patients provided informed consent. The study was conducted in accordance with the revised guidelines of the Declaration of Helsinki (2013).

From January 2022 to June 2024, we analyzed 1215 patients with thyroid nodules from four centers in China. The cohort included a training set (*n* = 524) and internal validation set (*n* = 225) from Changzhou First People’s Hospital, external validation set 1 (*n* = 51) from Suzhou Municipal Hospital, external validation set 2 (*n* = 174) from Shanghai Ruijin Hospital, and prospective external validation set 3 (*n* = 241) from Xuzhou Central Hospital (August 2024 to January 2025).

Inclusion criteria: age ≥ 18 years with pathologically confirmed classic PTC, complete clinical data, and high-quality preoperative CT and ultrasound images. Exclusion criteria: previous thyroid intervention, concomitant malignancy, poor-quality imaging, incomplete data, neoadjuvant therapy, non-curative surgery, or insufficient follow-up.

### Clinical data collection

Risk stratification was performed according to the 2015 ATA management guidelines [2]. For the purpose of model development, intermediate-risk and high-risk categories were combined into a single “intermediate/high-risk” group to optimize the binary classification performance and clinical decision-making utility, as both categories typically require active treatment rather than surveillance. Clinical variables included demographic data, laboratory parameters, and pathological findings from preoperative fine-needle aspiration biopsy (FNAB). Immunological markers, including platelet-to-lymphocyte ratio (PLR) and others, were calculated from routine blood tests obtained 3–5 days before surgery. Chronic lymphocytic thyroiditis (CLT) status was recorded based on preoperative assessment, including elevated serum anti-thyroid peroxidase antibodies and/or anti-thyroglobulin antibodies, and characteristic ultrasonographic features (diffuse hypoechogenicity and heterogeneous echotexture) [2]. For multifocal carcinomas, the number of tumor foci was determined by preoperative FNAB, and tumor location was defined by the lesion with the largest diameter. Pathological findings included cervical lymph node status and extrathyroidal extension. Multicollinearity analysis was performed using the variance inflation factor (VIF > 5 for exclusion). Multivariate logistic regression identified independent risk factors.

### Image acquisition and processing

CT image quality control: All CT scans were performed using standardized protocols across centers. For Siemens Somatom Definition Flash scanners (primary equipment), parameters were: 120 kV, 1.0–1.5 mm slice thickness, 512 × 512 matrix. Quality assessment criteria included: signal-to-noise ratio > 20, contrast-to-noise ratio > 3, and absence of motion artifacts.

Ultrasound image quality control: High-frequency transducers (5–15 MHz) from four manufacturers were used. Standardization protocol included: depth setting 2–4 cm, gain optimization for optimal tumor visualization, and focus positioned at tumor center. Quality assessment criteria: adequate tumor visualization in near and far fields, minimal acoustic shadowing, and clear tumor boundaries.

### Region of interest segmentation

Tumor regions were manually segmented by two experienced radiologists (more than 10 years’ experience) using 3D Slicer software (version 5.0). Ultrasound segmentation was performed on the largest cross-sectional area, while CT segmentation was performed on all tumor-containing slices to construct three-dimensional volumes. Inter-observer agreement was assessed using the intraclass correlation coefficient (ICC) on 150 randomly selected cases stratified across all four participating centers, with independent segmentations performed by both radiologists. ICC values demonstrated excellent agreement: 0.94 for ultrasound segmentation (95% CI: 0.89–0.96) and 0.92 for CT segmentation (95% CI: 0.88–0.94), both exceeding the predefined threshold of 0.85 for acceptable reproducibility.

### Ultrasound habitat imaging analysis

Ultrasound regions of interest (ROIs) underwent superpixel over-segmentation using K-means clustering. Four distinct habitats were identified using the Calinski-Harabasz index. Radiomic features were extracted using PyRadiomics (version 3.0.1). Feature selection used Mann–Whitney U test filtering, correlation filtering, and least absolute shrinkage and selection operator (LASSO) regularization with 10-fold cross-validation. Six machine learning algorithms were evaluated: Random Forest (RF), Gradient Boosting Machine (GBM), Convolutional Neural Network, K-Nearest Neighbors, Logistic Regression, and Support Vector Machine (SVM).

### CT habitat imaging analysis

CT images underwent supervoxel segmentation and two-stage clustering to identify five habitat subregions. Spatial heterogeneity was quantified using the Multi-Scale Index (MSI) matrix approach. Feature selection employed F-score ranking. The same six machine learning algorithms were applied.

### Multimodal fusion strategies

Three fusion approaches were implemented: early fusion (concatenating all features), late fusion (combining individual model predictions), and ensemble fusion (advanced ensemble techniques).

### Model evaluation and validation

Performance metrics included AUC, sensitivity, specificity, positive and negative predictive values, accuracy, F1 score, and Brier score. Decision curve analysis assessed clinical utility. Internal validation used 5-fold cross-validation. External validation was performed using three independent cohorts.

### Model interpretability and clinical implementation

SHAP analysis provided model interpretability. A web-based prediction system was developed using the Streamlit framework for real-time risk stratification.

### Statistical analysis

Statistical analyses were performed using R (version 4.2.1) and Python (version 3.8). Continuous variables were compared using *t*-test or Mann–Whitney U test. Categorical variables were analyzed using chi-square or Fisher’s exact test. Statistical significance was defined as *p* < 0.05. Model comparisons used DeLong’s test.

## Results

### Patient characteristics and study cohorts

**Fig. 1:**
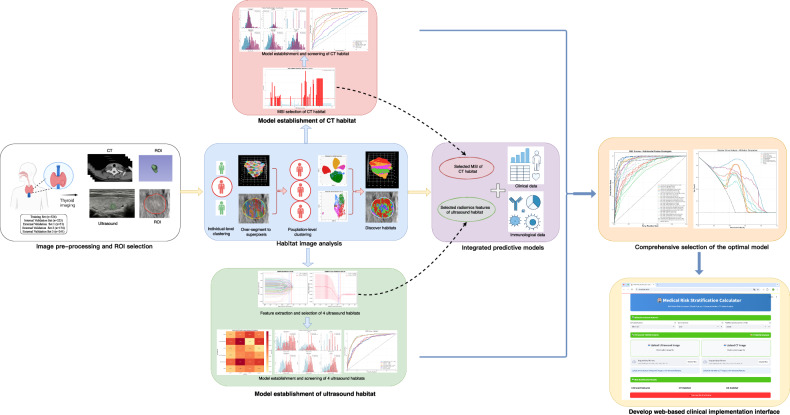
Study workflow and patient cohort characteristics. Overview of the multicenter study design showing patient recruitment from four institutions, data preprocessing pipeline, habitat imaging analysis framework, and model development strategy. The workflow demonstrates the integration of ultrasound and CT habitat analysis with clinical features for risk stratification in PTC patients

This multicenter study enrolled 1215 patients with PTC across four institutions. The study workflow is shown in Fig. 1.

Baseline characteristics are summarized in Table 1. Mean age ranged from 38.9 to 43.9 years across cohorts. BRAF V600E mutations were detected in 86.7 to 94.1% of patients. CLT was present in 20.9 to 29.4% of patients. Mean tumor diameter was 1.1 to 1.2 cm. Risk stratification showed low-risk patients in 50.2 to 66.7% of each cohort. These characteristics demonstrated comparable distributions across training and validation cohorts.

**Table 1:** Clinical characteristics of patients in training and validation sets

Clinical features	Training	Internal validation	External validation 1	External validation 2	External validation 3
	*N* = 524	*N* = 225	*N* = 51	*N* = 174	*N* = 241
Gender (male)	146 (27.9%)	51 (22.7%)	17 (33.3%)	84 (48.3%)	56 (23.2%)
Age (years)	43.9 ± 12.1	42.7 ± 11.6	41.7 ± 11.5	38.9 ± 12.2	43.6 ± 12.2
BMI (kg/m²)	24.3 ± 3.8	24.1 ± 3.8	23.8 ± 4.3	24.6 ± 3.3	23.4 ± 4.2
BRAF V600E (mutation)	469 (89.3%)	195 (86.7%)	48 (94.1%)	154 (88.5%)	221 (91.7%)
CLT (presence)	127 (24.2%)	47 (20.9%)	15 (29.4%)	38 (21.8%)	55 (22.8%)
Tumor diameter (cm)	1.1 ± 0.8	1.1 ± 0.8	1.1 ± 0.7	1.2 ± 0.7	1.1 ± 0.8
Number of lesions (≥ 2)	136 (26.0%)	63 (28.0%)	13 (25.5%)	71 (40.8%)	56 (23.2%)
Tumor location (middle/lower)	408 (77.9%)	176 (78.2%)	37 (72.5%)	129 (74.1%)	181 (75.1%)
Serum Tg (ng/mL)	34.2 ± 81.9	38.0 ± 80.2	25.6 ± 70.9	30.0 ± 63.3	29.9 ± 64.5
TgAb (IU/mL)	138.3 ± 466.7	89.3 ± 385.5	227.6 ± 711.5	150.5 ± 544.5	165.3 ± 573.4
TPOAb (IU/mL)	39.0 ± 88.2	32.3 ± 72.4	56.7 ± 108.4	51.7 ± 110.8	46.6 ± 99.0
LMR	5.5 ± 4.3	5.9 ± 4.4	4.1 ± 3.2	5.2 ± 4.3	6.3 ± 4.4
NLR	4.2 ± 2.7	4.3 ± 2.7	4.9 ± 2.9	5.1 ± 2.8	3.1 ± 2.1
PLR	238.1 ± 146.9	242.7 ± 144.7	270.3 ± 158.7	288.2 ± 144.3	180.9 ± 109.7
SII	1438.7 ± 1187.2	1467.8 ± 1153.0	1783.6 ± 1297.1	1870.9 ± 1176.9	897.7 ± 870.0
Risk stratification					
Low	276 (52.7%)	126 (56.0%)	34 (66.7%)	89 (51.1%)	121 (50.2%)
Intermediate/high	248 (47.3%)	99 (44.0%)	17 (33.3%)	85 (48.9%)	120 (49.8%)

*BMI* body mass index, *CLT* chronic lymphocytic thyroiditis, *TgAb* thyroglobulin antibody, *Tg* thyroglobulin, *TPOAb* peroxidase antibody, *LMR* lymphocyte to monocyte ratio, *NLR* neutrophil to lymphocyte ratio, *PLR* platelet-to lymphocyte ratio, *SII* systemic immune inflammation index

### Clinical risk factor analysis

Multicollinearity analysis was performed using VIF to assess feature independence. BMI demonstrated high multicollinearity (VIF = 7.05) and was excluded from further analysis. The remaining clinical features were subjected to multivariate logistic regression analysis.

Three variables emerged as independent risk factors for intermediate/high-risk stratification (Table 2). CLT showed a significant association with increased risk (odds ratio (OR) = 1.622, 95% confidence interval (CI): 1.070–2.457, *p* = 0.023). Tumor size demonstrated strong predictive value (OR = 1.554, 95% CI: 1.212–1.992, *p* = 0.001). Platelet-to-lymphocyte ratio (PLR) was identified as an independent predictor (OR = 1.602, 95% CI: 1.401–1.833, *p* = 0.004). These three factors were incorporated into subsequent multimodal model development.

**Table 2:** Collinearity and logistic regression analysis of clinical features associated with intermediate/high risk stratification

Clinical features	VIF	β	Odds ratio (95% CI)	*p*-value
Gender (male)	1.13	−0.218	0.804 (0.531–1.218)	0.304
Age (≥ 55 years)	1.04	−0.207	0.808 (0.534–1.224)	0.314
BMI (kg/m²)	7.05	N/A	N/A	N/A
BRAF V600E mutation	1.80	−0.065	0.937 (0.512–1.715)	0.834
CLT (presence)	1.64	0.484	1.622 (1.070–2.457)	0.023
Tumor size (cm)	1.24	0.441	1.554 (1.212–1.992)	0.001
Number of lesions = 2	1.10	−0.071	0.931 (0.584–1.485)	0.931
Number of lesions ≥ 3	1.06	0.746	2.108 (0.097–4.380)	0.056
Location (middle pole)	1.57	−0.399	0.671 (0.430–1.047)	0.079
Location (lower pole)	1.58	0.221	1.247 (0.723–2.151)	0.426
Serum thyroglobulin (ng/mL)	1.26	0.001	1.001 (0.999–1.003)	0.389
Thyroglobulin antibody (IU/mL)	1.17	0.000	1.000 (0.999–1.000)	0.475
Peroxidase antibody (IU/mL)	1.47	0.001	1.001 (0.998–1.003)	0.549
Lymphocyte to monocyte ratio	1.91	0.007	1.007 (0.959–1.057)	0.770
Neutrophil to lymphocyte ratio	2.42	−0.037	0.964 (0.81–1.131)	0.652
Platelet to lymphocyte ratio	1.31	0.469	1.602 (1.401–1.833)	0.004
SII	1.35	0.001	1.001 (0.999–1.002)	0.890

*VIF* variance inflation factor, *BMI* body mass index, *CLT* chronic lymphocytic thyroiditis, *SII* systemic immune inflammation index, *CI* confidence interval

### Ultrasound habitat imaging analysis

**Fig. 2:**
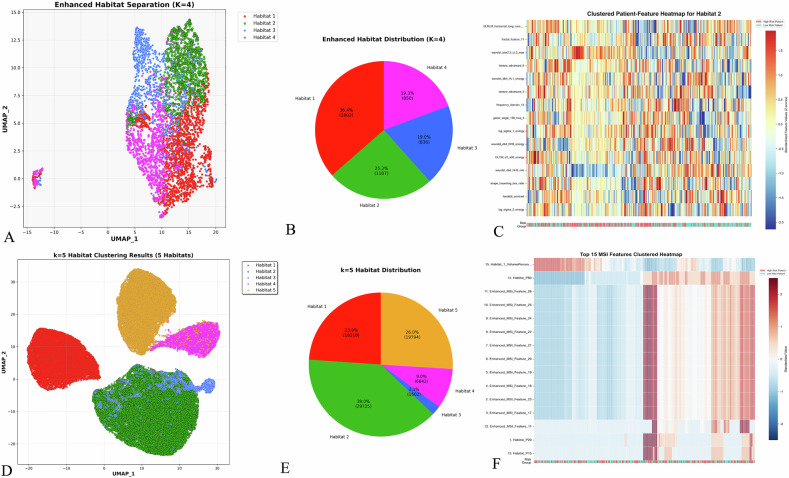
Habitat imaging analysis and feature extraction. **A**, **B** UMAP visualization and distribution of four ultrasound habitats identified through K-means clustering. **C** Clustered heatmap showing the top 15 discriminative features from ultrasound Habitat 2. **D**, **E** UMAP visualization and distribution of five CT habitats identified through two-stage clustering. **F** Clustered heatmap displaying the top 15 multi-scale index (MSI) features from CT habitat analysis

**Fig. 3:**
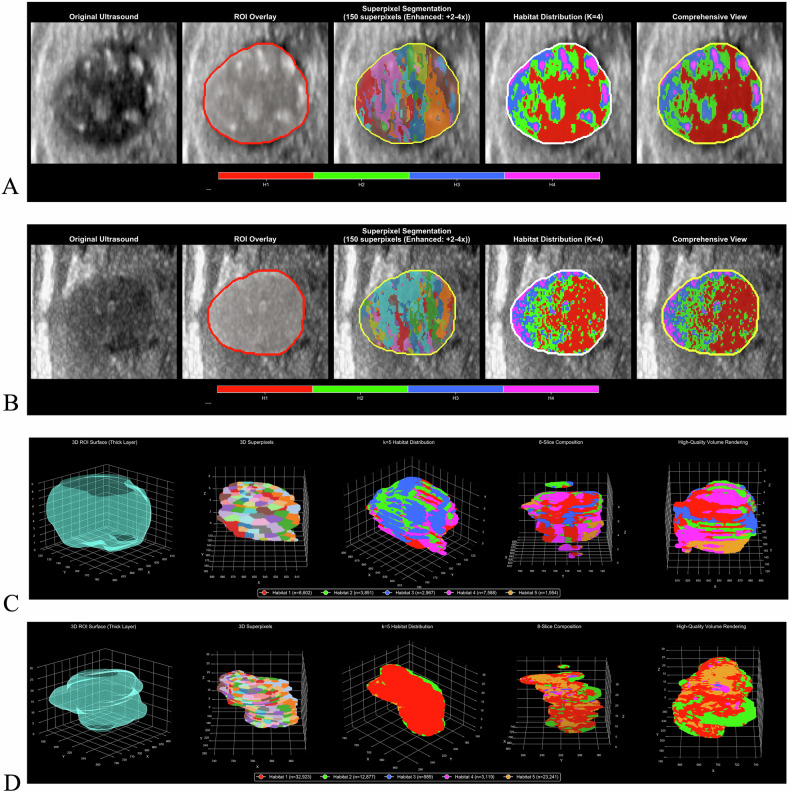
Representative habitat segmentation examples. **A** Ultrasound habitat segmentation example for intermediate/high-risk PTC patient, showing original image, ROI overlay, superpixel segmentation, habitat distribution, and comprehensive view. **B** Ultrasound habitat segmentation example for a low-risk PTC patient, demonstrating the same processing workflow. **C** Three-dimensional CT habitat visualization for intermediate/high-risk PTC patient, displaying ROI surface rendering, superpixel segmentation, habitat distribution, slice composition, and high-quality volume rendering. **D** Three-dimensional CT habitat visualization for a low-risk PTC patient, showing corresponding processing steps and habitat distribution patterns

Ultrasound regions of interest (ROIs) underwent superpixel over-segmentation using the K-means clustering algorithm. Four distinct habitats were identified and visualized using uniform manifold approximation and projection (UMAP) dimensionality reduction (Fig. 2A, B, Supplementary Fig. S1, Fig. 3A, B). Each representing specific biological characteristics: Habitat 1 (hyperechoic regions, 36.4%) corresponds to areas with low cellularity and potential fibrotic changes; Habitat 2 (hypoechoic heterogeneous regions, 25.2%) represents areas of high cellular density and proliferative activity, associated with aggressive tumor behavior; Habitat 3 (isoechoic regions, 19.0%) indicates moderate cellularity with preserved tissue architecture; and Habitat 4 (complex echogenic patterns, 19.3%) reflects areas of mixed composition including potential calcifications and necrotic changes.

A total of 635 ultrasound radiomic features were extracted from each habitat. Feature selection yielded 21 features for Habitat 1, 33 features for Habitat 2, 38 features for Habitat 3, and 30 features for Habitat 4 (Supplementary Fig. S2). Feature correlation matrices are presented in Supplementary Fig. S3.

Six machine learning algorithms were evaluated for each habitat (Supplementary Table S1). Habitat 2 demonstrated superior performance across all cohorts. In the training set, ultrasound (US)-Habitat 2-RF achieved the highest AUC of 0.92. This performance was maintained in internal validation (AUC = 0.89) and external validation cohorts (AUC = 0.80–0.92). Based on these results, Habitat 2 was designated as the high-risk habitat. The top 15 discriminative features from Habitat 2 are visualized in Fig. 2C.

### CT habitat imaging analysis

CT images underwent supervoxel segmentation and two-stage clustering to identify five distinct habitat subregions with specific pathophysiological significance based on multi-scale index (MSI) feature analysis (Fig. 2D, E, Supplementary Fig. S4, Fig. 3C, D): Habitat 1 (23.9%) represents core tumor regions with moderate inter-habitat interactions; Habitat 2 (39.0%) demonstrates the highest interaction complexity, indicating the most biologically active tumor areas with intense proliferation and tissue remodeling; Habitat 3 (2.1%) exhibits intermediate interactions, representing transition zones; Habitat 4 (9.0%) shows minimal interactions corresponding to low metabolic regions including necrotic and fibrotic areas; and Habitat 5 (26.0%) represents peripheral tumor regions with variable enhancement patterns.

MSI features were extracted to construct spatial interaction matrices for the five-habitat architecture. A total of 120 MSI features were derived to quantify spatial heterogeneity (Table 3). These features included second-order statistical measures from GLCM analysis, absolute habitat volumes (H1-H5), habitat-boundary interactions for each habitat, inter-habitat co-occurrence patterns between all habitat pairs, normalized spatial relationships, and advanced texture, morphological, and information-theoretic descriptors for comprehensive tumor architecture characterization.

**Table 3:** 120-dimensional multi-scale index feature documentation with grouped display

Feature_Range	Feature_Description	Methodology	Mean	Std
MSI 1–MSI 4	2nd-order statistical features derived from GLCM analysis of habitat texture patterns	GLCM computation with enhanced numerical stability	26.88	81.14
MSI 5–MSI 9	Tumor habitat volume (H1–H5) measured by direct voxel counting, i.e., MSI 5 = Vol(H1), MSI 6 = Vol(H2), MSI 7 = Vol(H3), MSI 8 = Vol(H4), MSI 9 = Vol(H5)	Direct voxel counting within optimized habitat clusters	8.45	15.32
MSI 10–MSI 14	Habitat-boundary interaction patterns for each habitat, i.e., MSI 10 = H1∩Border, MSI 11 = H2∩Border, MSI 12 = H3∩Border, MSI 13 = H4∩Border, MSI 14 = H5∩Border	Spatial adjacency analysis between habitats and tumor periphery	0.18	0.12
MSI 15–MSI 24	Inter-habitat co-occurrence patterns: H1 interactions (MSI 15–18, mean = 5.20), H2 interactions (MSI 19–21, mean = 103.28), H3 interactions (MSI 22–23, mean = 15.71), H4 ∩ H5 interaction (MSI 24, mean = 0.43)	Co-occurrence counting for all habitat pair combinations	31.15	81.24
MSI 25–MSI 29	Normalized habitat volumes as percentage of total tumor volume, i.e., MSI 25 = Vol(H1)/Vol(Total), MSI 26 = Vol(H2)/Vol(Total), MSI 27 = Vol(H3)/Vol(Total), MSI 28 = Vol(H4)/Vol(Total), MSI 29 = Vol(H5)/Vol(Total)	Habitat volume divided by total tumor volume	20.02	13.85
MSI 30–MSI 44	Normalized spatial interaction patterns: habitat-boundary interactions (MSI 30–34), H1 normalized interactions (MSI 35–38), H2 normalized interactions (MSI 39–41), H3 normalized interactions (MSI 42–43), H4 ∩ H5 normalized interactions (MSI 44)	All interaction values normalized by the total tumor volume	0.73	0.85
MSI 45–MSI 59	Advanced texture analysis features: Gabor filtering (MSI 45–49), wavelet energy (MSI 50–54), and local binary patterns (MSI 55–59) for each habitat, capturing multiscale texture information and directional patterns	Multi-resolution texture analysis with optimized filter banks	2.81	3.35
MSI 60–MSI 74	Morphological complexity features: surface area measurements (MSI 60–64), convexity analysis (MSI 65–69), and sphericity measurements (MSI 70–74) for each habitat, quantifying geometric properties	Comprehensive morphometric analysis of habitat geometry	0.50	0.23
MSI 75–MSI 94	Statistical distribution features: intensity means (MSI 75–79), standard deviations (MSI 80–84), skewness (MSI 85–89), and kurtosis (MSI 90–94) for each habitat, characterizing intensity distributions	Statistical moment computation within habitat regions	0.19	0.11
MSI 95–MSI 114	Information-theoretic and connectivity features: entropy measurements (MSI 95–99), fractal dimensions (MSI 100–104), connectivity analysis (MSI 105–109), and eccentricity measurements (MSI 110–114) for each habitat	Advanced complexity analysis combining information theory and graph theory	0.55	0.35
MSI 115–MSI 120	Global tumor architecture descriptors: comprehensive spatial heterogeneity features including global entropy, multiscale variance, pattern complexity, architectural index, tumor signature, and integrated heterogeneity measures	Multiscale complexity analysis combining geometric, statistical, and information-theoretic approaches	0.41	0.23

*MSI* multi-scale index, *GLCM* gray-level co-occurrence matrix, *H1-H5* Habitat 1-5, *Std* standard deviation, *Vol* volume, *LBP* local binary pattern

MSI features were extracted to construct spatial interaction matrices. A total of 120 MSI features were derived to quantify spatial heterogeneity (Table 3), including habitat volumes, inter-habitat interactions, boundary patterns, and advanced texture and morphological descriptors. Feature selection using F-score ranking (threshold > 1.0) identified 37 discriminative MSI features (Supplementary Fig. S5). The top 15 MSI features are visualized in Fig. 2F.

**Fig. 4:**
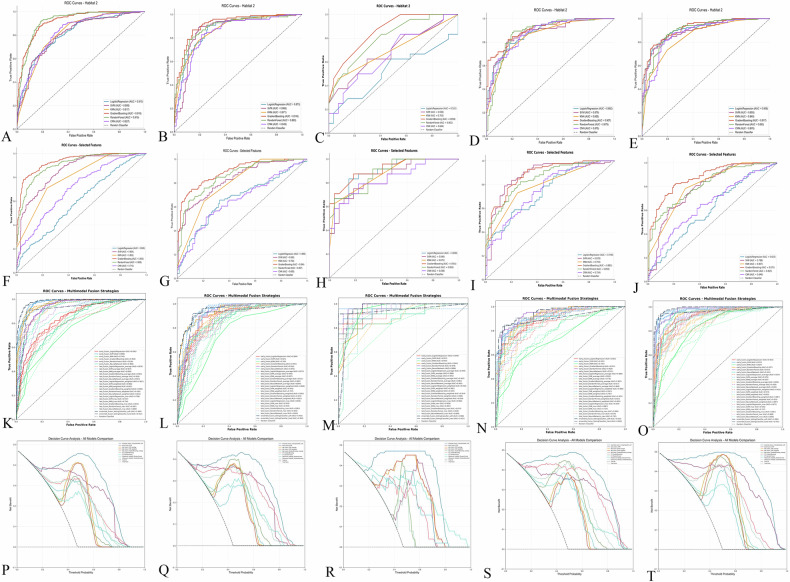
Feature selection pipeline and model performance evaluation. **A**–**E** Receiver operating characteristic performance comparison of six machine learning algorithms for ultrasound Habitat 2 across the training set, internal validation set, and three external validation sets. **F**–**J** Performance evaluation of six machine learning algorithms for CT habitat models across training set, internal validation set, and three external validation sets. **K**–**O** Comprehensive performance comparison of all fusion model strategies across the training set, internal validation set, and three external validation sets. **P**–**T** Decision curve analysis of nine high-performing models (US-habitat 2-RF, US-habitat 2-GBM, CT-habitat-RF, CT-habitat-GBM, Ensemble fusion Voting Classifier soft, Early fusion SVM, Late fusion SVM average, Late fusion SVM weighted, Late fusion GBM average) across training set, internal validation set, and three external validation sets

Six machine learning algorithms were evaluated on the selected MSI features. CT-habitat models demonstrated robust performance across validation cohorts (Fig. 4F–J, Supplementary Table S1). CT-habitat-GBM achieved the highest performance in the training set (AUC = 0.93) and maintained consistent results in validation cohorts (AUC = 0.88–0.92).

### Multimodal fusion model development

A comprehensive multimodal model was constructed by integrating clinical features (CLT, tumor size), immunological markers (PLR), ultrasound habitat 2 radiomic features, and CT-derived MSI features. Three fusion strategies were evaluated across all validation cohorts.

Performance evaluation is presented in Supplementary Table S2 and Fig. 4K–O. Early fusion demonstrated robust performance, with early_fusion_SVM achieving an AUC of 0.97 in the training set. Late fusion strategies showed variable performance depending on aggregation methods, with late_fusion_SVM_average and late_fusion_SVM_weighted achieving AUC values of 0.97. Ensemble fusion approaches exhibited superior performance, with ensemble_fusion_VotingClassifier_soft achieving an AUC of 0.98 in training and maintaining consistent performance across validation cohorts.

### Model performance comparison and optimal model selection

Nine high-performing models were selected for comprehensive comparison. Single-modality models included US-habitat 2-RF, US-habitat 2-GBM, CT-habitat-RF, and CT-habitat-GBM. Multimodal approaches comprised Ensemble fusion Voting Classifier soft, Early fusion SVM, Late fusion SVM average, Late fusion SVM weighted, and Late fusion GBM average.

Performance metrics across all validation cohorts are presented in Table 4. Receiver operating characteristic analysis and decision curve analysis revealed substantial differences between models (Fig. 4P–T). Single-modality models showed moderate performance, with AUC values ranging from 0.80 to 0.93 across validation sets. Multimodal fusion strategies demonstrated superior performance.

**Table 4:** Performance comparison of machine learning models for habitat classification across training, internal validation, and external validation sets

Models	Accuracy	AUC	Sensitivity	Specificity	PPV	NPV	F1 score	Brier score
Training set								
US-habitat 2-RF	0.85	0.92	0.88	0.83	0.82	0.88	0.85	0.15
US-habitat 2-GBM	0.83	0.92	0.81	0.86	0.83	0.83	0.82	0.17
CT-habitat-RF	0.83	0.91	0.78	0.87	0.84	0.81	0.81	0.17
CT-habitat-GBM	0.86	0.93	0.81	0.91	0.89	0.84	0.85	0.14
Ensemble fusion Voting Classifier soft	0.94	0.98	0.94	0.93	0.92	0.95	0.93	0.06
Early fusion SVM	0.90	0.97	0.93	0.88	0.88	0.93	0.90	0.10
Late fusion SVM average	0.92	0.97	0.93	0.91	0.90	0.93	0.91	0.08
Late fusion SVM weighted	0.91	0.97	0.92	0.91	0.90	0.93	0.91	0.09
Late fusion GBM average	0.89	0.97	0.81	0.95	0.94	0.85	0.87	0.11
Internal validation set								
US-habitat 2-RF	0.82	0.92	0.71	0.90	0.85	0.80	0.77	0.18
US-habitat 2-GBM	0.83	0.89	0.79	0.86	0.81	0.84	0.80	0.17
CT-habitat-RF	0.79	0.86	0.66	0.89	0.82	0.77	0.73	0.21
CT-habitat-GBM	0.82	0.89	0.65	0.95	0.91	0.77	0.76	0.18
Ensemble fusion Voting Classifier soft	0.88	0.95	0.90	0.87	0.85	0.92	0.87	0.12
Early fusion SVM	0.84	0.92	0.84	0.84	0.81	0.87	0.82	0.16
Late fusion SVM average	0.84	0.94	0.75	0.92	0.88	0.82	0.81	0.16
Late fusion SVM weighted	0.83	0.94	0.70	0.93	0.88	0.80	0.78	0.17
Late fusion GBM average	0.75	0.91	0.48	0.95	0.89	0.70	0.63	0.25
External validation set 1								
US-habitat 2-RF	0.69	0.80	0.83	0.56	0.63	0.79	0.71	0.31
US-habitat 2-GBM	0.75	0.86	0.58	0.89	0.82	0.71	0.68	0.25
CT-habitat-RF	0.71	0.88	0.71	0.70	0.68	0.73	0.69	0.29
CT-habitat-GBM	0.84	0.92	0.71	0.96	0.94	0.79	0.81	0.16
Ensemble fusion Voting Classifier soft	0.94	0.99	0.88	0.98	0.97	0.90	0.93	0.06
Early fusion SVM	0.87	0.96	0.83	0.89	0.87	0.86	0.85	0.13
Late fusion SVM average	0.86	0.96	0.83	0.89	0.87	0.86	0.85	0.13
Late fusion SVM weighted	0.96	0.98	0.96	0.96	0.96	0.96	0.96	0.07
Late fusion GBM average	0.69	0.96	0.33	0.97	0.95	0.63	0.50	0.31
External validation set 2								
US-habitat 2-RF	0.82	0.88	0.80	0.83	0.82	0.81	0.81	0.18
US-habitat 2-GBM	0.80	0.91	0.72	0.88	0.85	0.76	0.78	0.20
CT-habitat-RF	0.74	0.85	0.60	0.88	0.82	0.70	0.69	0.26
CT-habitat-GBM	0.75	0.88	0.66	0.84	0.80	0.72	0.72	0.25
Ensemble fusion Voting Classifier soft	0.89	0.95	0.85	0.92	0.91	0.86	0.88	0.11
Early fusion SVM	0.85	0.92	0.76	0.93	0.92	0.81	0.83	0.15
Late fusion SVM average	0.86	0.92	0.78	0.93	0.92	0.81	0.84	0.14
Late fusion SVM weighted	0.86	0.92	0.78	0.93	0.92	0.81	0.84	0.14
Late fusion GBM average	0.81	0.90	0.73	0.89	0.86	0.77	0.79	0.19
External validation set 3								
US-habitat 2-RF	0.83	0.89	0.86	0.81	0.82	0.85	0.84	0.17
US-habitat 2-GBM	0.83	0.92	0.78	0.88	0.87	0.80	0.82	0.17
CT-habitat-RF	0.71	0.81	0.77	0.65	0.69	0.74	0.72	0.29
CT-habitat-GBM	0.79	0.88	0.83	0.76	0.77	0.81	0.80	0.21
Ensemble fusion Voting Classifier soft	0.93	0.99	0.94	0.93	0.93	0.94	0.93	0.07
Early fusion SVM	0.93	0.97	0.90	0.97	0.96	0.91	0.93	0.07
Late fusion SVM average	0.93	0.98	0.91	0.94	0.94	0.91	0.92	0.07
Late fusion SVM weighted	0.93	0.97	0.91	0.94	0.94	0.91	0.92	0.07
Late fusion GBM average	0.80	0.89	0.80	0.80	0.80	0.80	0.80	0.20

*AUC* area under the curve, *PPV* positive predictive value, *NPV* negative predictive value, *RF* Random Forest, *GBM* gradient boosting machine, *SVM* support vector machine, *US* ultrasound, *CT* computed tomography

Ensemble fusion Voting Classifier soft emerged as the optimal model. This approach achieved the highest AUC values: 0.98 in training, 0.95 in internal validation, and 0.95–0.99 across external validation cohorts. The model maintained exceptional consistency with accuracy exceeding 0.88 in all validation sets. Brier scores indicated superior calibration, ranging from 0.06 to 0.12 across cohorts. Decision curve analysis confirmed substantial clinical benefit across the full range of threshold probabilities, validating its clinical utility for risk stratification.

### Model interpretability and clinical implementation

**Fig. 5:**
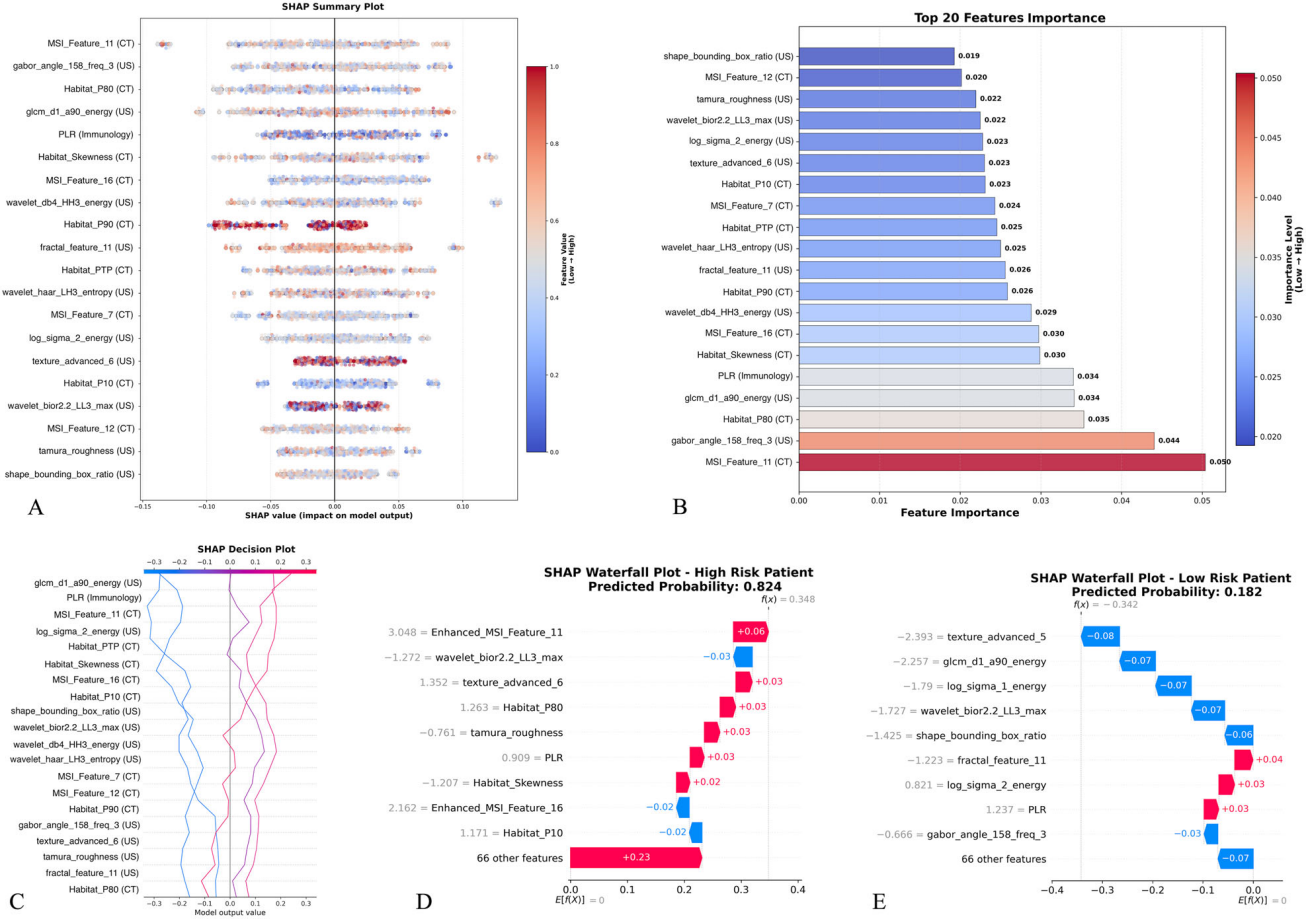
Model interpretability analysis using the SHAP framework. **A** SHAP summary plot showing global feature importance and impact directions for the optimal ensemble model, with MSI_Feature_11 (CT), gabor_angle_158_freq_3 (US), and Habitat_P80 (CT) as the most influential predictors. **B** Feature importance ranking of top 20 predictors, demonstrating multimodal integration with MSI_Feature_11 achieving the highest importance score (0.050), followed by gabor_angle_158_freq_3 (0.044) and Habitat_P80 (0.035). **C** SHAP decision plot illustrating model decision boundaries across different risk categories. **D**, **E** Individual patient explanations using SHAP waterfall plots for representative high-risk (predicted probability: 0.824) and low-risk (predicted probability: 0.182) cases, demonstrating feature contributions to final predictions

SHAP (SHapley Additive exPlanations) analysis was employed to provide comprehensive interpretability for the optimal Ensemble fusion Voting Classifier soft model (Fig. 5). Global feature importance analysis revealed a balanced contribution from multimodal features, with MSI_Feature_11 (CT) achieving the highest importance score (0.050), followed by gabor_angle_158_freq_3 (ultrasound) at 0.044. The top-ranking predictors demonstrated effective integration across imaging modalities, including CT habitat features (Habitat_P80), ultrasound texture features (glcm_d1_a90_energy), and immunological markers (PLR).

The decision plot analysis illustrated clear separation between risk categories based on cumulative SHAP values (Fig. 5C). Individual patient interpretability was demonstrated through SHAP waterfall plots for representative high-risk and low-risk cases (Fig. 5D, E). High-risk patients showed positive contributions from enhanced MSI features and specific habitat characteristics, while low-risk patients exhibited opposing feature patterns.

**Fig. 6:**
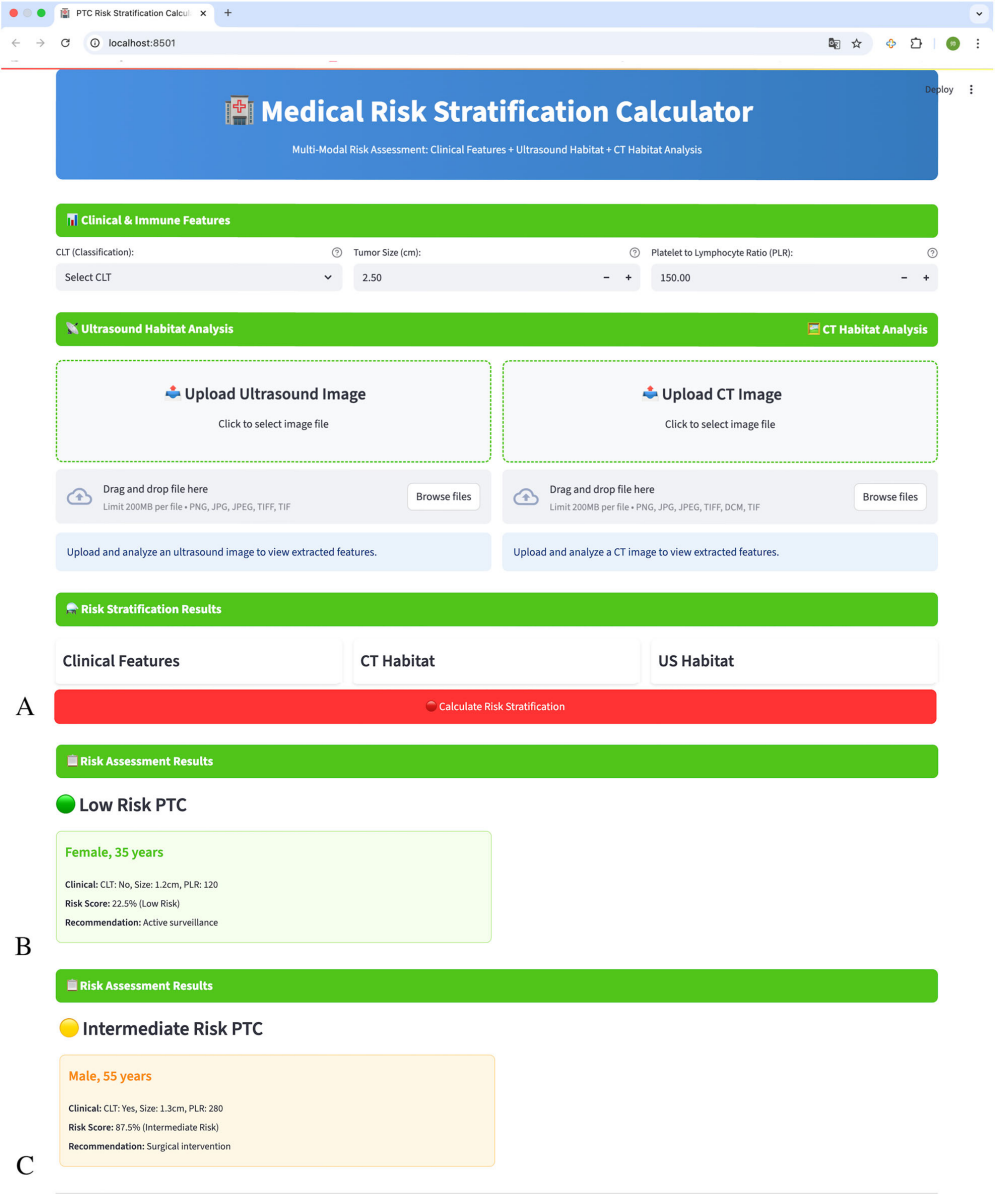
Web-based clinical implementation system. **A** User interface of the Medical Risk Stratification Calculator showing input sections for clinical features, ultrasound habitat analysis, and CT habitat analysis with drag-and-drop image upload functionality. **B**, **C** Example risk assessment results displaying low-risk (22.5% probability) and intermediate-risk (87.5% probability) stratification with corresponding clinical recommendations and patient demographic information

A web-based clinical implementation system was developed using the Streamlit framework (Fig. 6). The interactive platform enables real-time risk stratification through automated image upload and processing. Users can input clinical parameters and upload ultrasound and CT images for immediate risk assessment. The system provides comprehensive risk scores, risk stratification, and clinical recommendations. Example outputs demonstrate successful stratification of low-risk (22.5% probability) and intermediate-risk (87.5% probability) patients, facilitating clinical decision-making.

## Discussion

This study developed a novel preoperative risk stratification model for PTC using habitat imaging combined with multimodal analysis. Our approach successfully integrated CT-derived MSI features, ultrasound habitat characteristics, clinical parameters, and immunological markers to identify low-risk patients who may benefit from active surveillance rather than immediate surgical intervention.

Previous studies have explored radiomics applications in thyroid cancer, but the vast majority focused on conventional machine learning combined with radiomic feature extraction or deep learning methods for direct image analysis [6, 7]. Recent advances have primarily concentrated on diagnostic classification rather than preoperative risk stratification [8–10]. Yu et al [11] constructed a transfer learning radiomics model for predicting lymph node metastasis in PTC. Notably, habitat imaging studies specifically for thyroid cancer risk stratification are extremely rare, with only limited research, such as Tang et al [11] exploring single-modality ultrasound-based habitat radiomics models for predicting lymph node metastasis. To establish the clinical significance of our approach, we systematically compared our habitat imaging method against established preoperative risk stratification models. Li et al [5] developed PRAC-PTC using multidimensional machine learning (clinical, immunological, genetic, and proteomic features), achieving AUC values of 0.787–0.799 with 84.4% accuracy. Liu et al [12] reported a radiopathomics model integrating ultrasound and cytology images, achieving AUC values of 0.886–0.828. Within our study, single-modality models showed AUC ranges of 0.80–0.93 across validation cohorts (ultrasound habitat: 0.80–0.92; CT habitat: 0.88–0.92). In contrast, our optimal ensemble fusion model achieved an AUC of 0.95–0.99, representing a 6–19% improvement over single-modality approaches and 12–27% improvement over published multimodal models. This substantial performance gain, coupled with consistent accuracy exceeding 88% across all validation cohorts, demonstrates the clinical value of multimodal habitat integration for enhanced preoperative risk prediction accuracy. Our habitat imaging approach addresses these limitations by providing a comprehensive risk assessment based entirely on preoperative data, establishing a new benchmark for preoperative PTC risk stratification that significantly outperforms existing methodologies.

The habitat imaging framework differs fundamentally from traditional whole-tumor analysis by focusing on spatial heterogeneity-based tumor characterization. Our approach identified tumor subregions with distinct radiological signatures, where each habitat represents specific pathophysiological states, including varying degrees of cellularity, vascularization, necrosis, and metabolic activity [13]. CT-derived habitats primarily captured structural variations and density differences based on X-ray attenuation properties [14], while ultrasound habitats reflected acoustic properties and microvascular patterns through sound wave behavior in biological tissues [15]. The integration of CT-derived MSI features, ultrasound habitat characteristics, clinical parameters, and immunological markers achieved superior predictive accuracy for distinguishing low-risk from intermediate/high-risk PTC patients. This multimodal habitat approach provided enhanced spatial resolution of tumor heterogeneity and enabled more precise risk stratification compared to traditional single-modality analyses.

SHAP analysis identified the most discriminative features in our multimodal fusion model for PTC aggressiveness prediction. Among CT-derived features, MSI_Feature_11 achieved the paramount importance representing multiscale tumor habitat heterogeneity and spatial complexity patterns that characterize aggressive tumor biology [16], while Habitat_P80 quantifies high-density tissue distributions within tumor subregions, reflecting cellular proliferation and invasive potential [17]. For ultrasound-derived features, gabor_angle_158_freq_3 captured oriented texture gradients representing disrupted tissue architectural organization, whereas glcm_d1_a90_energy measured textural uniformity with decreased values indicating increased tumor heterogeneity and randomness characteristic of aggressive behavior [18]. PLR represented the most important immunological parameter. Elevated PLR reflects systemic inflammatory responses and compromised immune surveillance mechanisms [19]. This inflammatory-immunosuppressive state facilitates tumor progression and metastatic potential [20]. This multimodal approach integrates spatial architecture, tissue organization, and systemic inflammatory features for comprehensive aggressiveness prediction.

Based on our optimal fusion model screening, we developed a web-based prediction platform that integrates automated habitat segmentation and feature extraction capabilities. This platform enables clinicians to upload imaging data and automatically predict patient risk stratification as either low-risk or intermediate/high-risk categories. For low-risk patients identified by our model, active surveillance with regular ultrasound monitoring represents an appropriate management strategy, potentially avoiding unnecessary surgical procedures and associated complications [2, 21]. For intermediate/high-risk patients, surgical treatment is recommended, with adjuvant radioactive iodine therapy considered based on postoperative pathological findings [22, 23]. This platform particularly benefits resource-constrained healthcare settings, including primary care facilities and rural hospitals with limited endocrinological expertise [24–26].

This study has several limitations that should be acknowledged. The retrospective design limits the strength of evidence compared to prospective studies and introduces inherent selection bias. The habitat segmentation process requires manual tumor delineation, which may introduce operator-dependent variability and limit reproducibility across different institutions. The study population consisted entirely of Chinese patients from four institutions within a single country, which represents a significant limitation for global clinical applicability. This homogeneous ethnic composition may limit generalizability to Western populations, African populations, and other ethnic groups due to potential differences in tumor biology, genetic susceptibility patterns, imaging characteristics, and clinical presentation. The genetic and environmental factors that influence papillary thyroid carcinoma phenotypes may vary substantially across different ethnic backgrounds, potentially affecting the performance of our habitat imaging models. The imaging protocols varied across participating centers, and despite harmonization procedures, technical variability may affect feature stability and model performance. Furthermore, all participating institutions utilized similar imaging equipment and protocols typical of Chinese healthcare systems, which may not reflect the diversity of imaging technologies, acquisition parameters, and quality standards employed in Western healthcare institutions. These technical and procedural differences represent additional barriers to international generalizability. The biological correlation between imaging-derived habitat features and underlying pathological processes requires further validation through histopathological studies. We propose a systematic validation strategy including correlative histopathological analysis with immunohistochemical staining for key markers (Ki-67, CD31, VEGF), spatial transcriptomics analysis to map gene expression profiles within identified habitats, and longitudinal studies tracking habitat evolution during treatment response [27, 28].

Future research should focus on prospective validation in larger, multicenter cohorts with standardized imaging protocols to confirm the clinical utility of this approach. External validation in Western populations, multi-ethnic cohorts, and diverse healthcare systems is essential to establish the global applicability of our habitat imaging framework. Additionally, development and validation of automated segmentation algorithms using deep learning approaches, including 3D U-Net and transformer-based architectures, will be essential to eliminate manual delineation requirements and streamline clinical workflow for routine implementation. Integration with emerging biomarkers, such as molecular genetic profiles or circulating tumor DNA, could further enhance predictive accuracy [29, 30].

This study demonstrates that habitat imaging combined with multimodal analysis provides a powerful framework for preoperative risk stratification in PTC. The developed fusion model enables clinicians to make informed preoperative decisions and provide individualized treatment recommendations.

## Supplementary information

ELECTRONIC SUPPLEMENTARY MATERIAL

## Data Availability

The datasets, trained models, and code that support the findings of this study are available from the corresponding author upon reasonable request. The clinical datasets are not publicly available due to patient privacy protection and ethical restrictions in accordance with institutional review board requirements. However, the habitat imaging analysis pipeline, machine learning algorithms, and model architectures can be shared to facilitate reproducibility. Researchers interested in accessing the materials should contact the corresponding author with a detailed research proposal and appropriate data use agreements.

## Abbreviations

ATA: American Thyroid Association
AUC: Area under the curve
BMI: Body mass index
CLT: Chronic lymphocytic thyroiditis
CT: Computed tomography
FNAB: Fine-needle aspiration biopsy
GBM: Gradient boosting machine
GLCM: Gray-level co-occurrence matrix
ICC: Intraclass correlation coefficient
MSI: Multi-scale index
PLR: Platelet-to-lymphocyte ratio
PTC: Papillary thyroid carcinoma
RF: Random forest
ROI: Region of interest
SHAP: SHapley Additive exPlanations
SVM: Support vector machine
UMAP: Uniform manifold approximation and projection
US: Ultrasound
VIF: Variance inflation factor
