# Supplementary material for: Habitat imaging combined with multimodal analysis for preoperative risk stratification of papillary thyroid carcinoma
Source: Insights Imaging. 2025 Dec 2;16:268. doi: 10.1186/s13244-025-02145-9 (PMC12672970; doi:10.1186/s13244-025-02145-9)
Supplement: Supplementary file 1 — ELECTRONIC SUPPLEMENTARY MATERIAL [file 13244_2025_2145_MOESM1_ESM.pdf]

# Habitat Imaging Combined with Multimodal Analysis for Preoperative Risk Stratification of Papillary Thyroid Carcinoma

## ELECTRONIC SUPPLEMENTARY MATERIAL

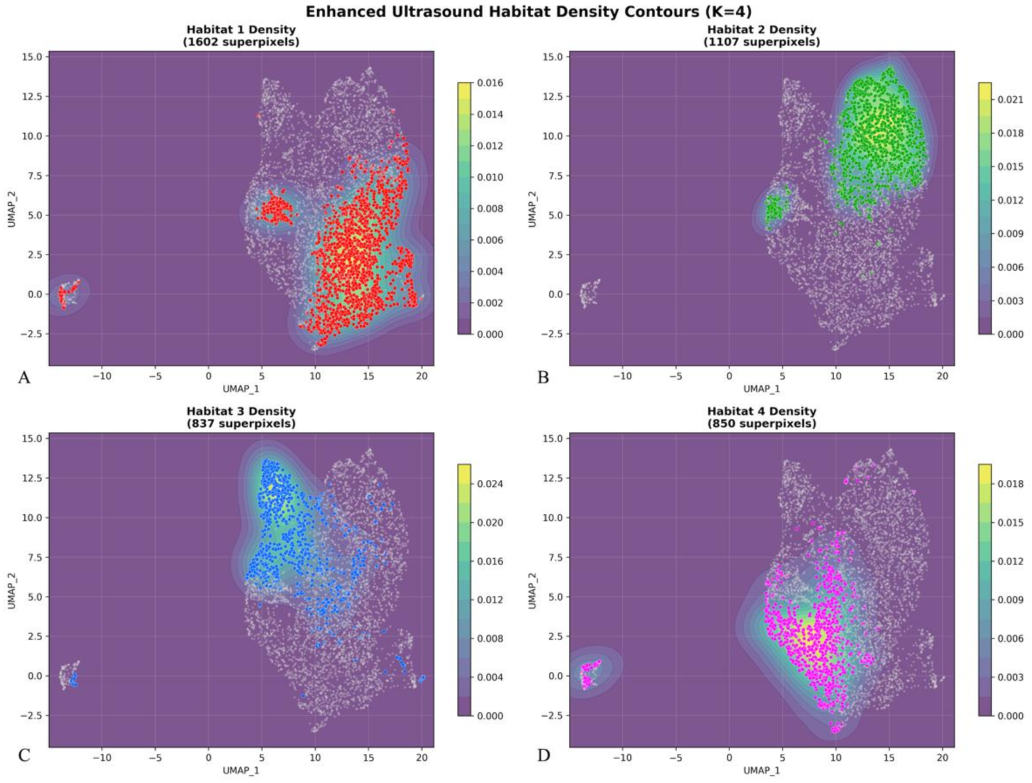


Supplementary Figure S1. Detailed UMAP visualization and density analysis of ultrasound habitats. (A-D) Detailed distribution and density visualization of four ultrasound habitats (Habitats 1-4) in UMAP dimensionality reduction space, showing spatial clustering patterns, superpixel density distributions, and corresponding superpixel counts (Habitat 1: 1,602 superpixels; Habitat 2: 1,107 superpixels; Habitat 3: 837 superpixels; Habitat 4: 850 superpixels), demonstrating unique distribution characteristics of different habitats in feature space.

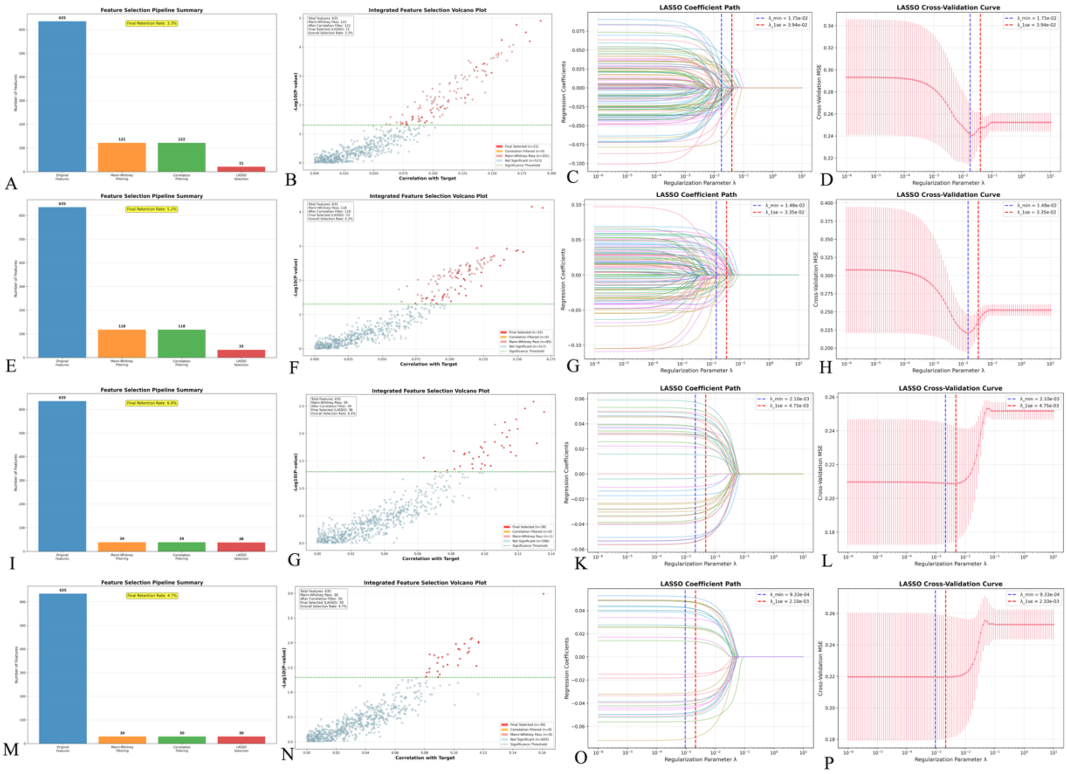


Supplementary Figure S2. Detailed sequential feature selection pipeline for ultrasound habitats. (A-D) Three-step feature selection process for ultrasound Habitat 1, reducing from 635 initial features to 21 final features, including Mann-Whitney filtering, correlation filtering volcano plot, LASSO regularization coefficient paths, and cross-validation curves. (E-H) Feature selection pipeline for ultrasound Habitat 2, reducing to 33 features. (I-L) Feature selection process for ultrasound Habitat 3, reducing to 38 features. (M-P) Feature selection workflow for ultrasound Habitat 4, reducing to 30 features. LASSO regularization for each habitat demonstrates dynamic changes in feature importance and determination of optimal feature subsets.

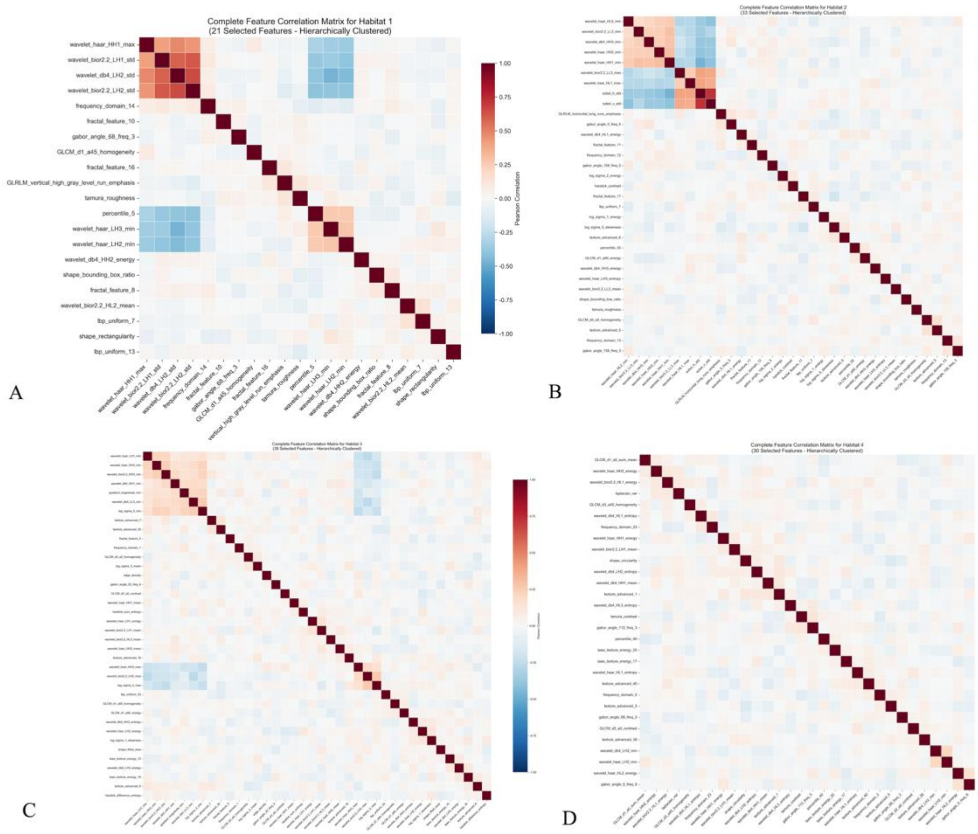


Supplementary Figure S3. Detailed analysis of ultrasound habitat feature correlation matrices. (A-D) Hierarchically clustered correlation heatmaps for final selected features from ultrasound Habitats 1-4, demonstrating correlation strength between features within each habitat model, clustering patterns, and feature independence validation. Heatmaps organize features using hierarchical clustering methods, with color intensity representing Pearson correlation coefficients, red indicating positive correlation, and blue indicating negative correlation. These matrices validate the effectiveness of the feature selection process, ensuring low redundancy and high independence of model input features.

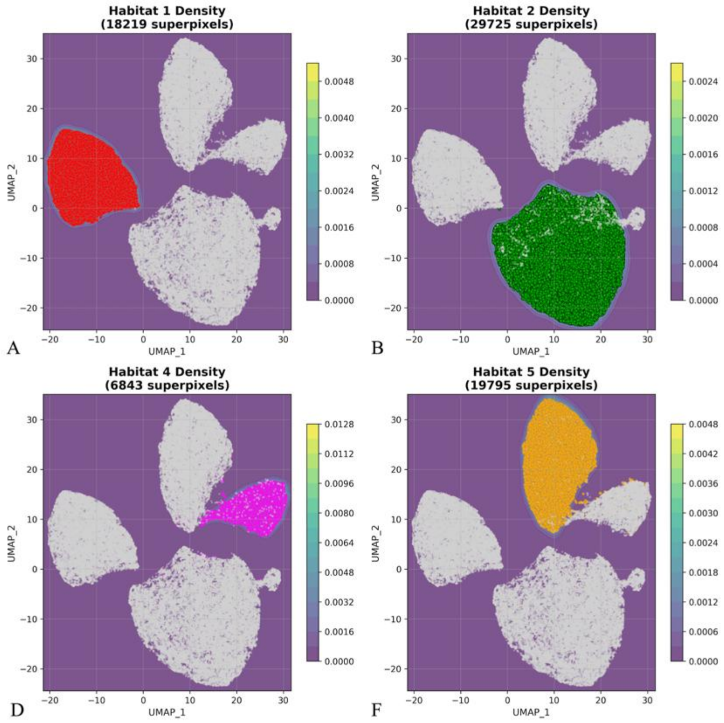


Supplementary Figure S4. Detailed UMAP visualization and clustering validation of CT habitats. Detailed UMAP dimensionality reduction visualization for CT habitat identification, showing distribution patterns, clustering boundaries, and spatial organization of five CT habitat subregions in feature space, with corresponding superpixel counts (Habitat 1: 18,219 superpixels; Habitat 2: 29,725 superpixels; Habitat 3: 1,602 superpixels; Habitat 4: 6,843 superpixels; Habitat 5: 19,795 superpixels). The image demonstrates the effectiveness of the two-stage clustering method, with each habitat showing clear separation and internal consistency in UMAP space, validating the stability of clustering algorithms and biological significance of habitat definitions.

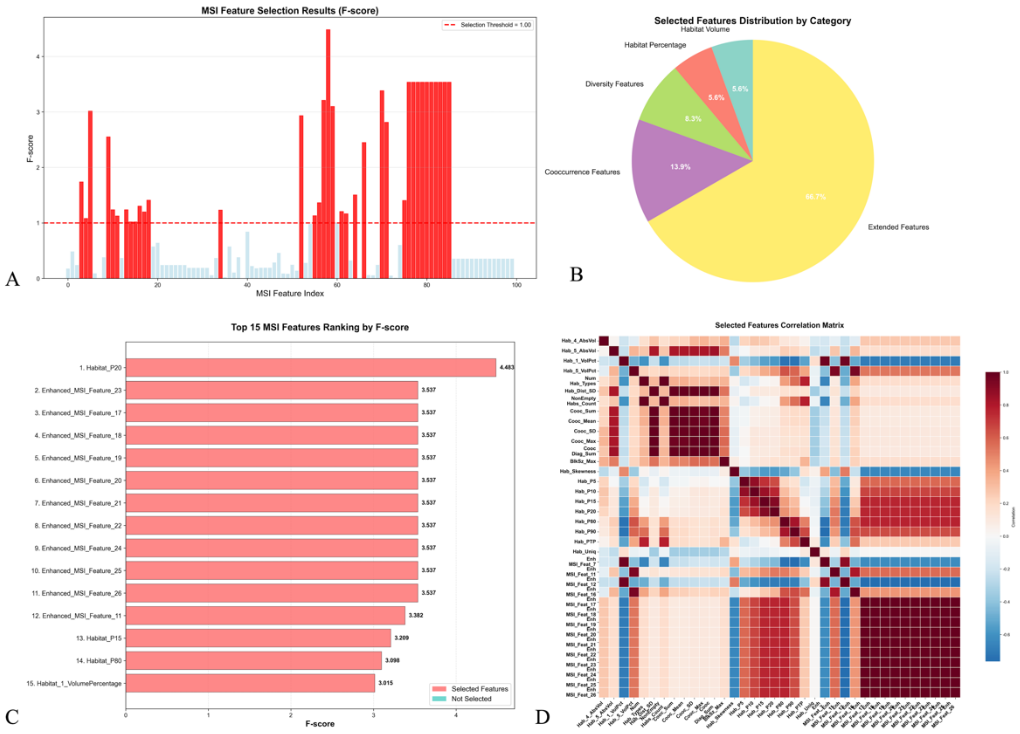


Supplementary Figure S5. Comprehensive selection and analysis of multi-scale index (MSI) features. (A) F-score distribution histogram for all 120 MSI features, showing selection threshold (F-score > 1.0) and feature filtering results, with 37 highly discriminative features finally selected. (B) Functional category distribution pie chart of selected features, showing relative proportions of extended features (67.6%), habitat volume (8.1%), co-occurrence features (13.5%), diversity features (5.4%), and other features (5.4%). (C) Top 15 MSI features ranked by F-score, with Habitat_P90 and Enhanced_MSI_Feature series dominating. (D) Correlation matrix heatmap of 37 selected MSI features, demonstrating independence between features and potential functional clustering patterns, validating the effectiveness of feature selection and diversity of model inputs.

Supplementary Table S1. Comprehensive performance comparison of individual machine learning models for habitat classification across training, internal validation, and external validation sets

| Models | Accuracy | AUC | Sensitivity | Specificity | PPV | NPV | F1 score |
|---|---|---|---|---|---|---|---|
| Training set | | | | | | | |
| US-habitat 2-RF | 0.85 | 0.92 | 0.88 | 0.83 | 0.82 | 0.88 | 0.85 |
| US-habitat 2-GBM | 0.83 | 0.92 | 0.81 | 0.86 | 0.83 | 0.83 | 0.82 |
| US-habitat 2-CNN | 0.76 | 0.83 | 0.82 | 0.70 | 0.71 | 0.81 | 0.76 |
| US-habitat 2-KNN | 0.75 | 0.82 | 0.69 | 0.79 | 0.75 | 0.74 | 0.72 |
| US-habitat 2-LR | 0.73 | 0.81 | 0.76 | 0.70 | 0.69 | 0.77 | 0.73 |
| US-habitat 2-SVM | 0.73 | 0.81 | 0.79 | 0.69 | 0.69 | 0.78 | 0.74 |
| CT-habitat-RF | 0.83 | 0.91 | 0.78 | 0.87 | 0.84 | 0.81 | 0.81 |
| CT-habitat-GBM | 0.86 | 0.93 | 0.81 | 0.91 | 0.89 | 0.84 | 0.85 |
| CT-habitat-CNN | 0.64 | 0.71 | 0.64 | 0.64 | 0.62 | 0.66 | 0.63 |
| CT-habitat-KNN | 0.74 | 0.80 | 0.71 | 0.76 | 0.73 | 0.75 | 0.72 |
| CT-habitat-LR | 0.55 | 0.60 | 0.48 | 0.62 | 0.53 | 0.57 | 0.51 |
| CT-habitat-SVM | 0.76 | 0.85 | 0.78 | 0.74 | 0.73 | 0.79 | 0.75 |
| Internal validation set | | | | | | | |
| US-habitat 2-RF | 0.83 | 0.89 | 0.79 | 0.86 | 0.81 | 0.84 | 0.80 |
| US-habitat 2-GBM | 0.82 | 0.92 | 0.71 | 0.90 | 0.85 | 0.80 | 0.77 |
| US-habitat 2-CNN | 0.78 | 0.85 | 0.74 | 0.81 | 0.75 | 0.80 | 0.74 |
| US-habitat 2-KNN | 0.80 | 0.87 | 0.78 | 0.81 | 0.76 | 0.82 | 0.77 |
| US-habitat 2-LR | 0.80 | 0.87 | 0.84 | 0.76 | 0.73 | 0.86 | 0.78 |
| US-habitat 2-SVM | 0.82 | 0.87 | 0.90 | 0.76 | 0.75 | 0.91 | 0.82 |
| CT-habitat-RF | 0.79 | 0.86 | 0.66 | 0.89 | 0.82 | 0.77 | 0.73 |
| CT-habitat-GBM | 0.82 | 0.89 | 0.65 | 0.95 | 0.91 | 0.77 | 0.76 |
| CT-habitat-CNN | 0.62 | 0.65 | 0.31 | 0.87 | 0.65 | 0.62 | 0.42 |
| CT-habitat-KNN | 0.73 | 0.79 | 0.65 | 0.79 | 0.71 | 0.74 | 0.68 |
| CT-habitat-LR | 0.64 | 0.67 | 0.60 | 0.68 | 0.60 | 0.68 | 0.60 |
| CT-habitat-SVM | 0.73 | 0.83 | 0.70 | 0.76 | 0.70 | 0.76 | 0.70 |
| External validation set 1 | | | | | | | |
| US-habitat 2-RF | 0.69 | 0.80 | 0.83 | 0.56 | 0.63 | 0.79 | 0.71 |
| US-habitat 2-GBM | 0.75 | 0.86 | 0.58 | 0.89 | 0.82 | 0.71 | 0.68 |
| US-habitat 2-CNN | 0.65 | 0.66 | 0.50 | 0.78 | 0.67 | 0.64 | 0.57 |
| US-habitat 2-KNN | 0.72 | 0.70 | 0.58 | 0.82 | 0.78 | 0.65 | 0.66 |
| US-habitat 2-LR | 0.45 | 0.52 | 0.38 | 0.52 | 0.41 | 0.48 | 0.39 |
| US-habitat 2-SVM | 0.67 | 0.70 | 0.63 | 0.70 | 0.65 | 0.68 | 0.64 |
| CT-habitat-RF | 0.71 | 0.88 | 0.71 | 0.70 | 0.68 | 0.73 | 0.69 |
| CT-habitat-GBM | 0.84 | 0.92 | 0.71 | 0.96 | 0.94 | 0.79 | 0.81 |
| CT-habitat-CNN | 0.78 | 0.84 | 0.71 | 0.85 | 0.81 | 0.77 | 0.76 |
| CT-habitat-KNN | 0.80 | 0.88 | 0.63 | 0.96 | 0.94 | 0.74 | 0.75 |
| CT-habitat-LR | 0.82 | 0.90 | 0.79 | 0.85 | 0.83 | 0.82 | 0.81 |
| CT-habitat-SVM | 0.76 | 0.85 | 0.67 | 0.85 | 0.80 | 0.74 | 0.73 |

| | | | | | | | |
|---|---|---|---|---|---|---|---|
| External validation set 2 | | | | | | | |
| US-habitat 2-RF | 0.82 | 0.88 | 0.80 | 0.83 | 0.82 | 0.81 | 0.81 |
| US-habitat 2-GBM | 0.80 | 0.91 | 0.72 | 0.88 | 0.85 | 0.76 | 0.78 |
| US-habitat 2-CNN | 0.76 | 0.88 | 0.75 | 0.78 | 0.76 | 0.77 | 0.76 |
| US-habitat 2-KNN | 0.78 | 0.85 | 0.71 | 0.85 | 0.82 | 0.75 | 0.76 |
| US-habitat 2-LR | 0.80 | 0.88 | 0.79 | 0.82 | 0.81 | 0.80 | 0.80 |
| US-habitat 2-SVM | 0.82 | 0.88 | 0.80 | 0.83 | 0.82 | 0.81 | 0.81 |
| CT-habitat-RF | 0.74 | 0.85 | 0.60 | 0.88 | 0.82 | 0.70 | 0.69 |
| CT-habitat-GBM | 0.75 | 0.88 | 0.66 | 0.84 | 0.80 | 0.72 | 0.72 |
| CT-habitat-CNN | 0.66 | 0.75 | 0.46 | 0.85 | 0.75 | 0.62 | 0.57 |
| CT-habitat-KNN | 0.74 | 0.79 | 0.73 | 0.75 | 0.74 | 0.74 | 0.74 |
| CT-habitat-LR | 0.64 | 0.75 | 0.66 | 0.62 | 0.62 | 0.65 | 0.64 |
| CT-habitat-SVM | 0.80 | 0.88 | 0.80 | 0.80 | 0.79 | 0.81 | 0.80 |
| External validation set 3 | | | | | | | |
| US-habitat 2-RF | 0.83 | 0.89 | 0.86 | 0.81 | 0.82 | 0.85 | 0.84 |
| US-habitat 2-GBM | 0.83 | 0.92 | 0.78 | 0.88 | 0.87 | 0.80 | 0.82 |
| US-habitat 2-CNN | 0.78 | 0.88 | 0.89 | 0.68 | 0.73 | 0.86 | 0.80 |
| US-habitat 2-KNN | 0.77 | 0.85 | 0.73 | 0.80 | 0.79 | 0.75 | 0.76 |
| US-habitat 2-LR | 0.82 | 0.91 | 0.83 | 0.81 | 0.81 | 0.83 | 0.82 |
| US-habitat 2-SVM | 0.83 | 0.89 | 0.83 | 0.83 | 0.83 | 0.83 | 0.83 |
| CT-habitat-RF | 0.71 | 0.81 | 0.77 | 0.65 | 0.69 | 0.74 | 0.72 |
| CT-habitat-GBM | 0.79 | 0.88 | 0.83 | 0.76 | 0.77 | 0.81 | 0.80 |
| CT-habitat-CNN | 0.57 | 0.65 | 0.73 | 0.42 | 0.55 | 0.61 | 0.63 |
| CT-habitat-KNN | 0.75 | 0.81 | 0.78 | 0.71 | 0.73 | 0.77 | 0.76 |
| CT-habitat-LR | 0.54 | 0.60 | 0.54 | 0.55 | 0.54 | 0.55 | 0.54 |
| CT-habitat-SVM | 0.73 | 0.79 | 0.80 | 0.66 | 0.70 | 0.77 | 0.75 |

*AUC* Area Under the Curve; *PPV* Positive Predictive Value; *NPV* Negative Predictive Value; *US* Ultrasound; *CT* Computed Tomography; *RF* Random Forest; *GBM* Gradient Boosting Machine; *CNN* Convolutional Neural Network; *KNN* K-Nearest Neighbors; *LR* Logistic Regression; *SVM* Support Vector Machine.

Supplementary Table S2. Detailed performance comparison of fusion strategies and machine learning models for habitat classification across training, internal validation, and external validation sets

| Models | Accuracy | AUC | Sensitivity | Specificity | PPV | NPV | F1 score |
|---|---|---|---|---|---|---|---|
| Training set | | | | | | | |
| early_fusion_GBM | 0.88 | 0.96 | 0.86 | 0.90 | 0.88 | 0.88 | 0.87 |
| early_fusion_KNN | 0.67 | 0.74 | 0.51 | 0.81 | 0.71 | 0.65 | 0.59 |
| early_fusion_LR | 0.75 | 0.85 | 0.75 | 0.76 | 0.74 | 0.77 | 0.74 |
| early_fusion_NN | 0.73 | 0.81 | 0.68 | 0.77 | 0.73 | 0.73 | 0.70 |
| early_fusion_ RF | 0.85 | 0.93 | 0.87 | 0.84 | 0.83 | 0.88 | 0.85 |
| early_fusion_SVM | 0.90 | 0.97 | 0.93 | 0.88 | 0.88 | 0.93 | 0.90 |
| ensemble_fusion_VotingClassifier_hard | 0.91 | 0.91 | 0.91 | 0.92 | 0.91 | 0.92 | 0.91 |
| ensemble_fusion_VotingClassifier_soft | 0.94 | 0.98 | 0.94 | 0.93 | 0.92 | 0.95 | 0.93 |
| late_fusion_GBM_average | 0.89 | 0.97 | 0.81 | 0.95 | 0.94 | 0.85 | 0.87 |
| late_fusion_GBM_max | 0.78 | 0.93 | 0.97 | 0.61 | 0.69 | 0.96 | 0.81 |
| late_fusion_GBM_weighted | 0.88 | 0.96 | 0.80 | 0.95 | 0.94 | 0.84 | 0.86 |
| late_fusion_KNN_average | 0.71 | 0.81 | 0.60 | 0.80 | 0.73 | 0.69 | 0.66 |
| late_fusion_KNN_max | 0.62 | 0.76 | 0.96 | 0.32 | 0.56 | 0.89 | 0.70 |
| late_fusion_KNN_weighted | 0.70 | 0.80 | 0.57 | 0.83 | 0.75 | 0.68 | 0.65 |
| late_fusion_LR_average | 0.74 | 0.83 | 0.75 | 0.74 | 0.72 | 0.76 | 0.73 |
| late_fusion_LR_max | 0.59 | 0.80 | 0.95 | 0.27 | 0.54 | 0.86 | 0.69 |
| late_fusion_LR_weighted | 0.75 | 0.83 | 0.75 | 0.75 | 0.73 | 0.77 | 0.74 |
| late_fusion_NN_average | 0.83 | 0.92 | 0.81 | 0.85 | 0.83 | 0.83 | 0.82 |
| late_fusion_NN_max | 0.68 | 0.88 | 0.97 | 0.41 | 0.60 | 0.94 | 0.74 |
| late_fusion_NN_weighted | 0.82 | 0.91 | 0.80 | 0.84 | 0.82 | 0.82 | 0.81 |
| late_fusion_RF_average | 0.85 | 0.94 | 0.81 | 0.89 | 0.87 | 0.84 | 0.84 |
| late_fusion_RF_max | 0.72 | 0.89 | 0.96 | 0.49 | 0.63 | 0.94 | 0.76 |
| late_fusion_RF_weighted | 0.85 | 0.94 | 0.79 | 0.89 | 0.87 | 0.83 | 0.83 |
| late_fusion_SVM_average | 0.92 | 0.97 | 0.93 | 0.91 | 0.90 | 0.93 | 0.91 |
| late_fusion_SVM_max | 0.59 | 0.95 | 0.98 | 0.23 | 0.53 | 0.94 | 0.69 |
| late_fusion_SVM_weighted | 0.91 | 0.97 | 0.92 | 0.91 | 0.90 | 0.93 | 0.91 |
| Internal validation set | | | | | | | |
| early_fusion_GBM | 0.87 | 0.94 | 0.77 | 0.94 | 0.92 | 0.84 | 0.84 |
| early_fusion_KNN | 0.65 | 0.75 | 0.44 | 0.81 | 0.65 | 0.65 | 0.53 |
| early_fusion_LR | 0.83 | 0.89 | 0.89 | 0.78 | 0.76 | 0.90 | 0.82 |
| early_fusion_NN | 0.80 | 0.87 | 0.68 | 0.90 | 0.84 | 0.78 | 0.75 |
| early_fusion_RF | 0.80 | 0.92 | 0.75 | 0.85 | 0.80 | 0.81 | 0.77 |
| early_fusion_SVM | 0.84 | 0.92 | 0.84 | 0.84 | 0.81 | 0.87 | 0.82 |
| ensemble_fusion_VotingClassifier_hard | 0.90 | 0.90 | 0.92 | 0.88 | 0.86 | 0.93 | 0.89 |
| ensemble_fusion_VotingClassifier_soft | 0.88 | 0.95 | 0.90 | 0.87 | 0.85 | 0.92 | 0.87 |
| late_fusion_GBM_average | 0.75 | 0.91 | 0.48 | 0.95 | 0.89 | 0.70 | 0.63 |
| late_fusion_GBM_max | 0.76 | 0.88 | 0.93 | 0.62 | 0.66 | 0.92 | 0.77 |
| late_fusion_GBM_weighted | 0.76 | 0.90 | 0.48 | 0.97 | 0.92 | 0.71 | 0.64 |
| late_fusion_KNN_average | 0.67 | 0.81 | 0.42 | 0.86 | 0.70 | 0.65 | 0.53 |

| | | | | | | | |
|---|---|---|---|---|---|---|---|
| late_fusion_KNN_max | 0.73 | 0.82 | 0.93 | 0.57 | 0.63 | 0.91 | 0.75 |
| late_fusion_KNN_weighted | 0.67 | 0.80 | 0.41 | 0.87 | 0.72 | 0.65 | 0.53 |
| late_fusion_LR_average | 0.79 | 0.88 | 0.83 | 0.75 | 0.73 | 0.85 | 0.77 |
| late_fusion_LR_max | 0.68 | 0.86 | 0.98 | 0.44 | 0.58 | 0.96 | 0.73 |
| late_fusion_LR_weighted | 0.78 | 0.87 | 0.81 | 0.75 | 0.72 | 0.83 | 0.76 |
| late_fusion_NN_average | 0.76 | 0.86 | 0.58 | 0.90 | 0.81 | 0.73 | 0.67 |
| late_fusion_NN_max | 0.72 | 0.84 | 0.95 | 0.54 | 0.62 | 0.93 | 0.75 |
| late_fusion_NN_weighted | 0.72 | 0.85 | 0.51 | 0.88 | 0.77 | 0.69 | 0.61 |
| late_fusion_RF_average | 0.78 | 0.89 | 0.70 | 0.85 | 0.78 | 0.78 | 0.74 |
| late_fusion_RF_max | 0.69 | 0.84 | 0.94 | 0.49 | 0.59 | 0.91 | 0.73 |
| late_fusion_RF_weighted | 0.76 | 0.87 | 0.67 | 0.84 | 0.77 | 0.76 | 0.71 |
| late_fusion_SVM_average | 0.84 | 0.94 | 0.75 | 0.92 | 0.88 | 0.82 | 0.81 |
| late_fusion_SVM_max | 0.80 | 0.94 | 0.95 | 0.64 | 0.69 | 0.94 | 0.81 |
| late_fusion_SVM_weighted | 0.83 | 0.94 | 0.70 | 0.93 | 0.88 | 0.80 | 0.78 |
| External validation set 1 | | | | | | | |
| early_fusion_GBM | 0.82 | 0.94 | 0.67 | 0.96 | 0.94 | 0.76 | 0.78 |
| early_fusion_KNN | 0.75 | 0.94 | 0.46 | 0.96 | 0.92 | 0.68 | 0.63 |
| early_fusion_LR | 0.86 | 0.95 | 0.83 | 0.89 | 0.87 | 0.86 | 0.85 |
| early_fusion_NN | 0.88 | 0.90 | 0.75 | 0.96 | 0.95 | 0.82 | 0.86 |
| early_fusion_RF | 0.92 | 0.98 | 0.92 | 0.93 | 0.92 | 0.93 | 0.92 |
| early_fusion_SVM | 0.87 | 0.96 | 0.83 | 0.89 | 0.87 | 0.86 | 0.85 |
| ensemble_fusion_VotingClassifier_hard | 0.92 | 0.92 | 0.92 | 0.93 | 0.92 | 0.93 | 0.92 |
| ensemble_fusion_VotingClassifier_soft | 0.94 | 0.99 | 0.88 | 0.98 | 0.97 | 0.90 | 0.93 |
| late_fusion_GBM_average | 0.69 | 0.96 | 0.33 | 0.97 | 0.95 | 0.63 | 0.50 |
| late_fusion_GBM_max | 0.73 | 0.87 | 0.83 | 0.63 | 0.67 | 0.81 | 0.74 |
| late_fusion_GBM_weighted | 0.71 | 0.95 | 0.38 | 0.96 | 0.91 | 0.64 | 0.55 |
| late_fusion_KNN_average | 0.86 | 0.93 | 0.71 | 0.96 | 0.94 | 0.79 | 0.83 |
| late_fusion_KNN_max | 0.61 | 0.80 | 0.96 | 0.30 | 0.55 | 0.89 | 0.70 |
| late_fusion_KNN_weighted | 0.71 | 0.92 | 0.38 | 0.96 | 0.91 | 0.64 | 0.55 |
| late_fusion_LR_average | 0.86 | 0.95 | 0.83 | 0.89 | 0.87 | 0.86 | 0.85 |
| late_fusion_LR_max | 0.73 | 0.97 | 0.92 | 0.48 | 0.63 | 0.87 | 0.77 |
| late_fusion_LR_weighted | 0.80 | 0.92 | 0.79 | 0.81 | 0.79 | 0.81 | 0.79 |
| late_fusion_NN_average | 0.86 | 0.96 | 0.88 | 0.85 | 0.84 | 0.88 | 0.86 |
| late_fusion_NN_max | 0.71 | 0.89 | 0.92 | 0.44 | 0.62 | 0.85 | 0.76 |
| late_fusion_NN_weighted | 0.86 | 0.96 | 0.88 | 0.85 | 0.84 | 0.88 | 0.86 |
| late_fusion_RF_average | 0.94 | 0.98 | 0.92 | 0.96 | 0.96 | 0.93 | 0.94 |
| late_fusion_RF_max | 0.73 | 0.93 | 0.92 | 0.48 | 0.63 | 0.87 | 0.77 |
| late_fusion_RF_weighted | 0.90 | 0.97 | 0.92 | 0.89 | 0.88 | 0.92 | 0.90 |
| late_fusion_SVM_average | 0.86 | 0.96 | 0.83 | 0.89 | 0.87 | 0.86 | 0.85 |
| late_fusion_SVM_max | 0.82 | 0.96 | 0.96 | 0.70 | 0.74 | 0.95 | 0.84 |
| late_fusion_SVM_weighted | 0.96 | 0.98 | 0.96 | 0.96 | 0.96 | 0.96 | 0.96 |
| External validation set 2 | | | | | | | |
| early_fusion_GBM | 0.84 | 0.92 | 0.80 | 0.88 | 0.86 | 0.82 | 0.83 |
| early_fusion_KNN | 0.67 | 0.73 | 0.46 | 0.87 | 0.76 | 0.63 | 0.57 |

| | | | | | | | |
|---|---|---|---|---|---|---|---|
| early_fusion_LR | 0.80 | 0.90 | 0.78 | 0.83 | 0.81 | 0.80 | 0.80 |
| early_fusion_NN | 0.84 | 0.91 | 0.82 | 0.85 | 0.84 | 0.84 | 0.83 |
| early_fusion_RF | 0.85 | 0.93 | 0.74 | 0.96 | 0.94 | 0.79 | 0.83 |
| early_fusion_SVM | 0.85 | 0.92 | 0.76 | 0.93 | 0.92 | 0.81 | 0.83 |
| ensemble_fusion_VotingClassifier_hard | 0.89 | 0.89 | 0.84 | 0.94 | 0.93 | 0.86 | 0.88 |
| ensemble_fusion_VotingClassifier_soft | 0.89 | 0.95 | 0.85 | 0.92 | 0.91 | 0.86 | 0.88 |
| late_fusion_GBM_average | 0.81 | 0.90 | 0.73 | 0.89 | 0.86 | 0.77 | 0.79 |
| late_fusion_GBM_max | 0.76 | 0.87 | 0.96 | 0.56 | 0.68 | 0.94 | 0.80 |
| late_fusion_GBM_weighted | 0.80 | 0.90 | 0.69 | 0.90 | 0.87 | 0.75 | 0.77 |
| late_fusion_KNN_average | 0.71 | 0.78 | 0.56 | 0.84 | 0.77 | 0.67 | 0.65 |
| late_fusion_KNN_max | 0.59 | 0.72 | 0.86 | 0.34 | 0.55 | 0.71 | 0.67 |
| late_fusion_KNN_weighted | 0.67 | 0.77 | 0.52 | 0.82 | 0.73 | 0.64 | 0.61 |
| late_fusion_LR_average | 0.80 | 0.89 | 0.79 | 0.82 | 0.81 | 0.80 | 0.80 |
| late_fusion_LR_max | 0.63 | 0.83 | 0.91 | 0.37 | 0.58 | 0.80 | 0.71 |
| late_fusion_LR_weighted | 0.79 | 0.88 | 0.76 | 0.82 | 0.80 | 0.78 | 0.78 |
| late_fusion_NN_average | 0.77 | 0.87 | 0.73 | 0.81 | 0.78 | 0.76 | 0.76 |
| late_fusion_NN_max | 0.67 | 0.79 | 0.88 | 0.47 | 0.61 | 0.81 | 0.72 |
| late_fusion_NN_weighted | 0.77 | 0.87 | 0.72 | 0.82 | 0.79 | 0.75 | 0.75 |
| late_fusion_RF_average | 0.84 | 0.92 | 0.80 | 0.89 | 0.87 | 0.82 | 0.83 |
| late_fusion_RF_max | 0.76 | 0.88 | 0.94 | 0.60 | 0.69 | 0.91 | 0.80 |
| late_fusion_RF_weighted | 0.84 | 0.91 | 0.79 | 0.89 | 0.87 | 0.81 | 0.83 |
| late_fusion_SVM_average | 0.86 | 0.92 | 0.78 | 0.93 | 0.92 | 0.81 | 0.84 |
| late_fusion_SVM_max | 0.78 | 0.89 | 0.89 | 0.66 | 0.72 | 0.87 | 0.80 |
| late_fusion_SVM_weighted | 0.86 | 0.92 | 0.78 | 0.93 | 0.92 | 0.81 | 0.84 |
| External validation set 3 | | | | | | | |
| early_fusion_GBM | 0.85 | 0.94 | 0.83 | 0.88 | 0.87 | 0.84 | 0.85 |
| early_fusion_KNN | 0.73 | 0.82 | 0.69 | 0.78 | 0.75 | 0.72 | 0.72 |
| early_fusion_LR | 0.83 | 0.92 | 0.83 | 0.83 | 0.83 | 0.83 | 0.83 |
| early_fusion_NN | 0.88 | 0.93 | 0.88 | 0.87 | 0.87 | 0.88 | 0.88 |
| early_fusion_RF | 0.89 | 0.97 | 0.94 | 0.83 | 0.85 | 0.94 | 0.89 |
| early_fusion_SVM | 0.93 | 0.97 | 0.90 | 0.97 | 0.96 | 0.91 | 0.93 |
| ensemble_fusion_VotingClassifier_hard | 0.94 | 0.94 | 0.94 | 0.94 | 0.94 | 0.94 | 0.94 |
| ensemble_fusion_VotingClassifier_soft | 0.93 | 0.99 | 0.94 | 0.93 | 0.93 | 0.94 | 0.93 |
| late_fusion_GBM_average | 0.80 | 0.89 | 0.80 | 0.80 | 0.80 | 0.80 | 0.80 |
| late_fusion_GBM_max | 0.65 | 0.84 | 0.92 | 0.31 | 0.59 | 0.79 | 0.74 |
| late_fusion_GBM_weighted | 0.78 | 0.89 | 0.78 | 0.79 | 0.78 | 0.79 | 0.78 |
| late_fusion_KNN_average | 0.72 | 0.82 | 0.73 | 0.72 | 0.72 | 0.73 | 0.72 |
| late_fusion_KNN_max | 0.59 | 0.75 | 0.95 | 0.21 | 0.55 | 0.80 | 0.70 |
| late_fusion_KNN_weighted | 0.71 | 0.80 | 0.71 | 0.71 | 0.71 | 0.71 | 0.71 |
| late_fusion_LR_average | 0.83 | 0.91 | 0.82 | 0.83 | 0.83 | 0.82 | 0.82 |
| late_fusion_LR_max | 0.65 | 0.87 | 0.95 | 0.32 | 0.59 | 0.86 | 0.73 |
| late_fusion_LR_weighted | 0.81 | 0.91 | 0.80 | 0.83 | 0.82 | 0.81 | 0.81 |
| late_fusion_NN_average | 0.81 | 0.90 | 0.73 | 0.90 | 0.88 | 0.77 | 0.79 |
| late_fusion_NN_max | 0.68 | 0.86 | 0.92 | 0.45 | 0.63 | 0.85 | 0.74 |

| | | | | | | | |
|---|---|---|---|---|---|---|---|
| late_fusion_NN_weighted | 0.82 | 0.90 | 0.74 | 0.89 | 0.87 | 0.78 | 0.80 |
| late_fusion_RF_average | 0.86 | 0.94 | 0.89 | 0.83 | 0.84 | 0.89 | 0.87 |
| late_fusion_RF_max | 0.65 | 0.89 | 0.95 | 0.31 | 0.59 | 0.89 | 0.74 |
| late_fusion_RF_weighted | 0.86 | 0.93 | 0.90 | 0.83 | 0.84 | 0.89 | 0.87 |
| late_fusion_SVM_average | 0.93 | 0.98 | 0.91 | 0.94 | 0.94 | 0.91 | 0.92 |
| late_fusion_SVM_max | 0.60 | 0.94 | 0.95 | 0.22 | 0.56 | 0.82 | 0.71 |
| late_fusion_SVM_weighted | 0.93 | 0.97 | 0.91 | 0.94 | 0.94 | 0.91 | 0.92 |

*AUC* Area Under the Curve; *PPV* Positive Predictive Value; *NPV* Negative Predictive Value; *GBM* Gradient Boosting Machine; *KNN* K-Nearest Neighbors; *LR* Logistic Regression; *NN* Neural Network; *RF* Random Forest; *SVM* Support Vector Machine.
